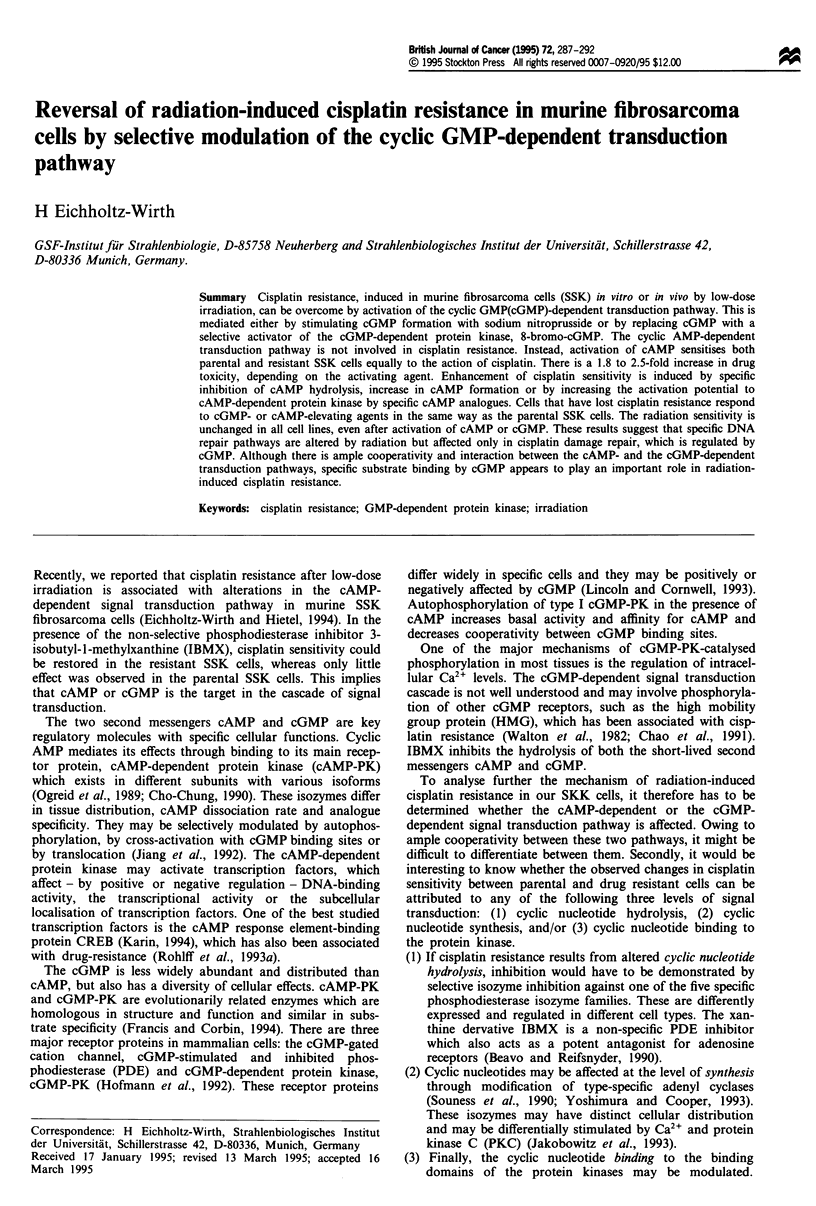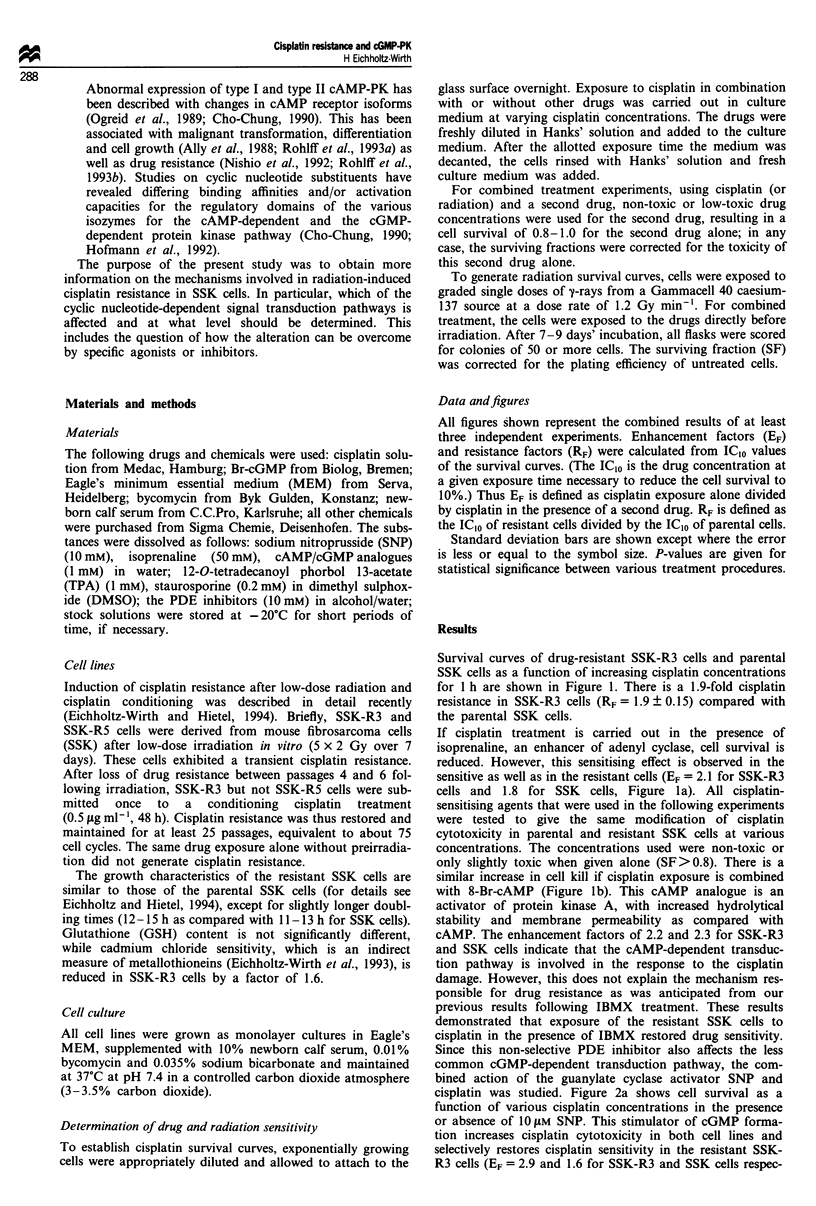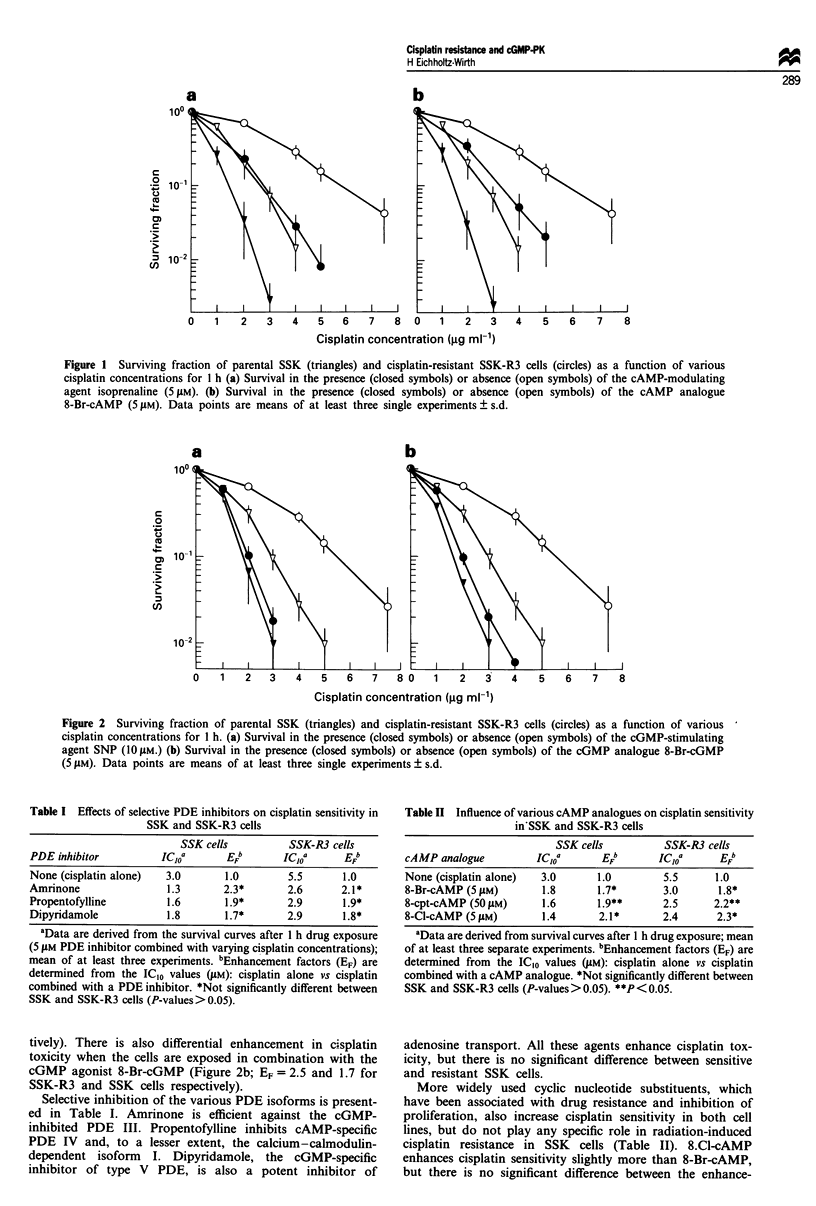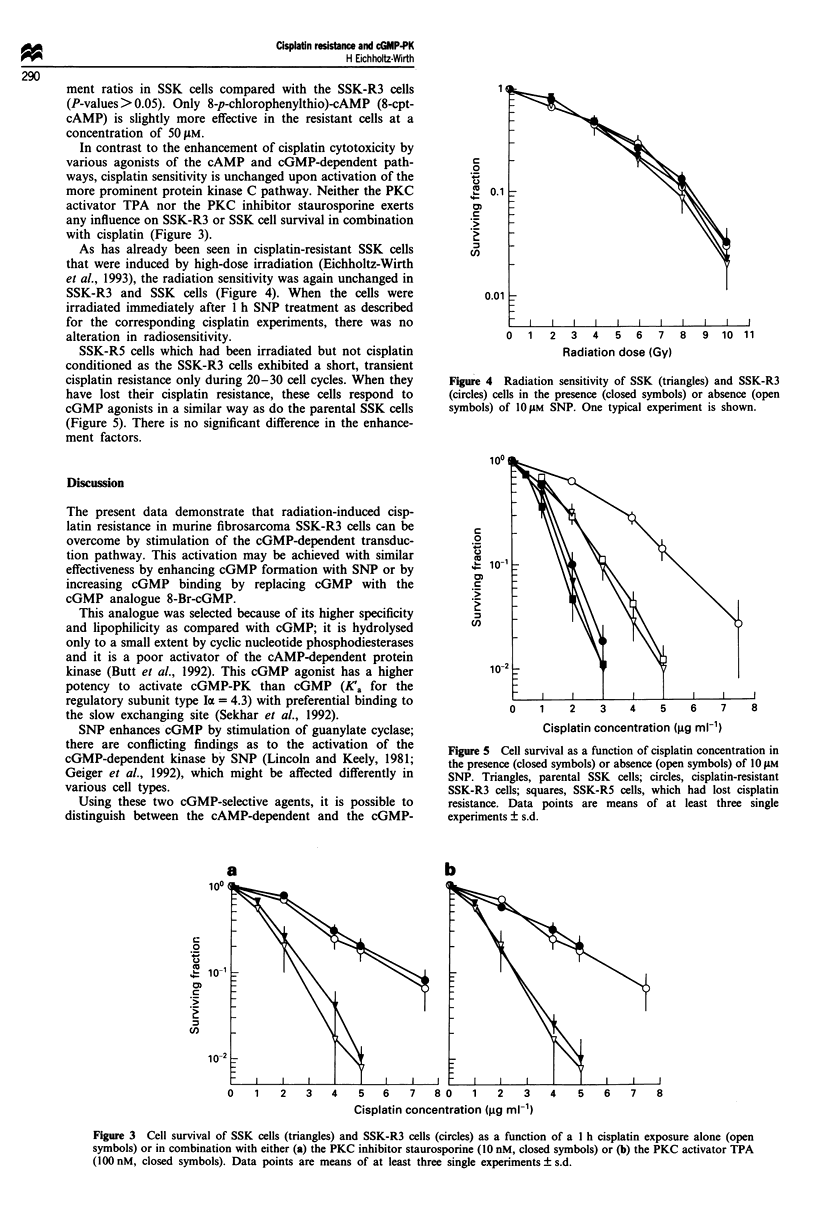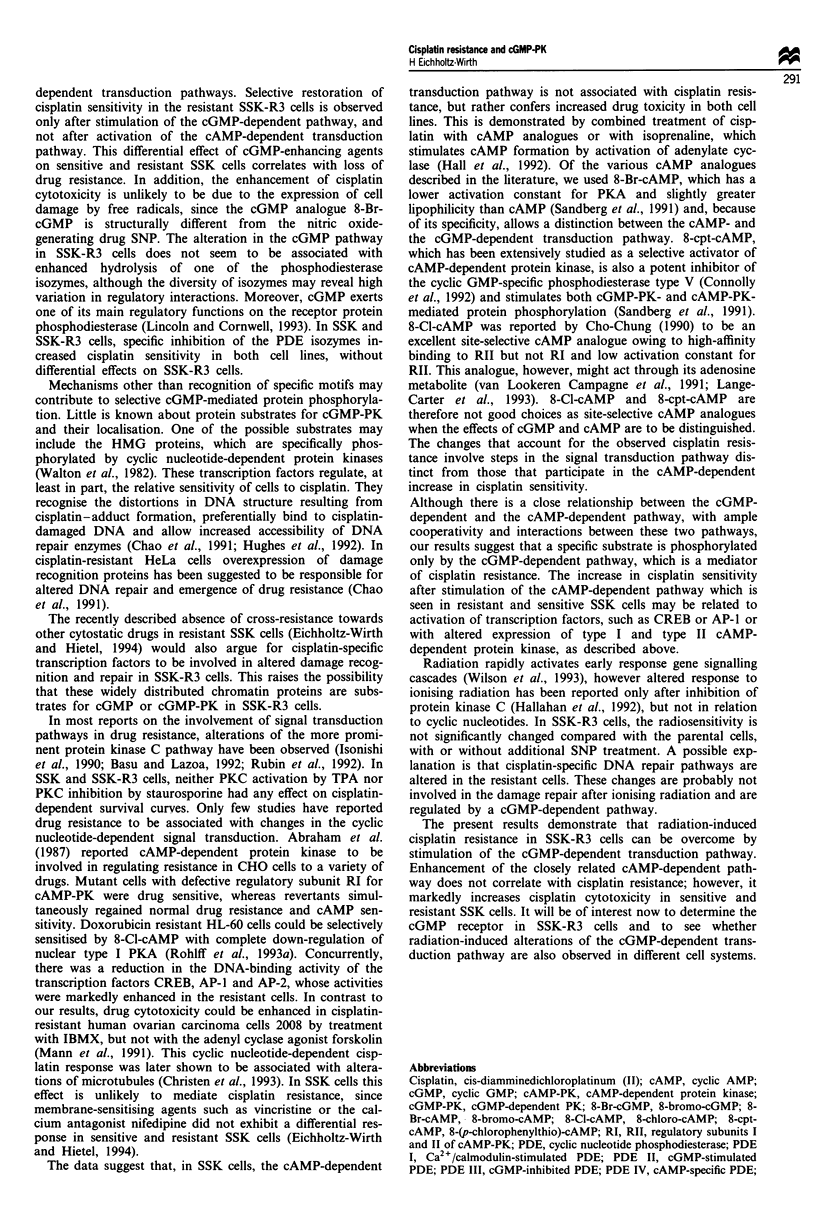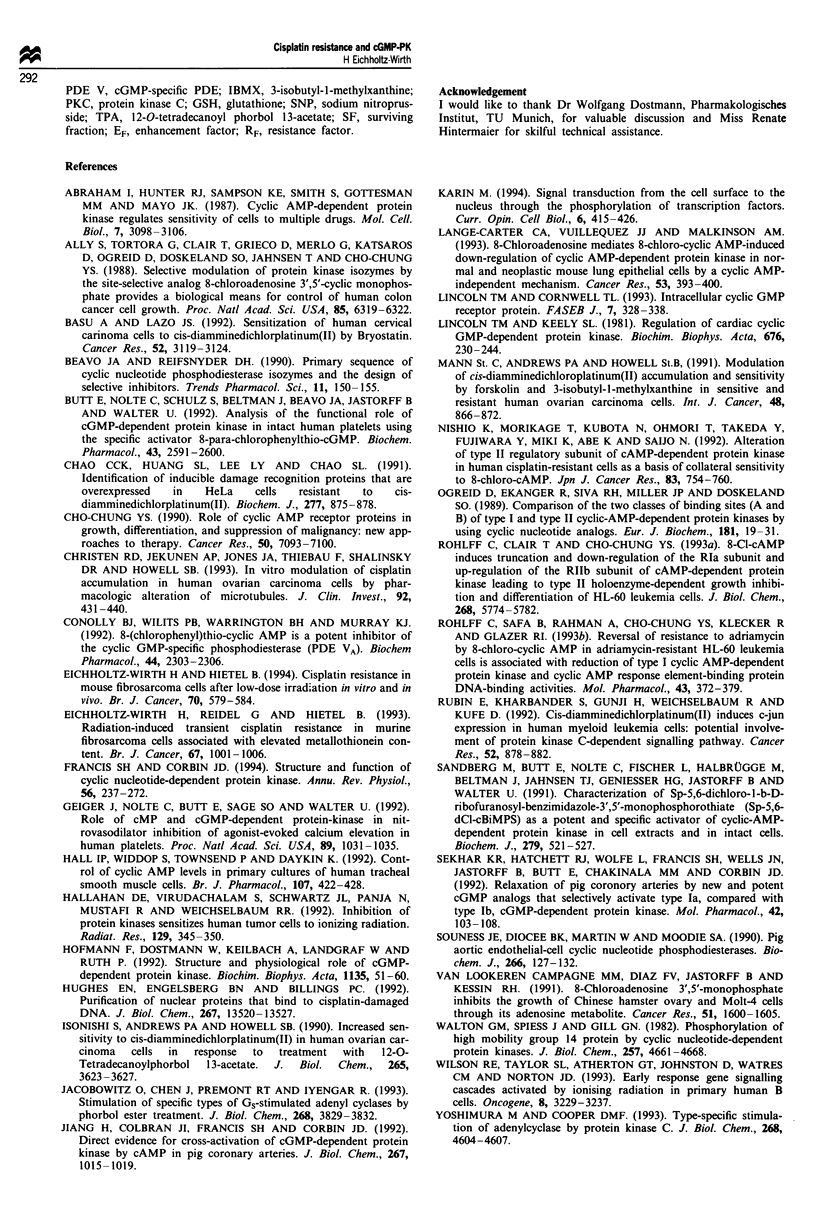# Reversal of radiation-induced cisplatin resistance in murine fibrosarcoma cells by selective modulation of the cyclic GMP-dependent transduction pathway.

**DOI:** 10.1038/bjc.1995.326

**Published:** 1995-08

**Authors:** H. Eichholtz-Wirth

**Affiliations:** GSF-Institut für Strahlenbiologie, Neuherberg, Germany.

## Abstract

Cisplatin resistance, induced in murine fibrosarcoma cells (SSK) in vitro or in vivo by low-dose irradiation, can be overcome by activation of the cyclic GMP(cGMP)-dependent transduction pathway. This is mediated either by stimulating cGMP formation with sodium nitroprusside or by replacing cGMP with a selective activator of the cGMP-dependent protein kinase, 8-bromo-cGMP. The cyclic AMP-dependent transduction pathway is not involved in cisplatin resistance. Instead, activation of cAMP sensitises both parental and resistant SSK cells equally to the action of cisplatin. There is a 1.8 to 2.5-fold increase in drug toxicity, depending on the activating agent. Enhancement of cisplatin sensitivity is induced by specific inhibition of cAMP hydrolysis, increase in cAMP formation or by increasing the activation potential to cAMP-dependent protein kinase by specific cAMP analogues. Cells that have lost cisplatin resistance respond to cGMP- or cAMP-elevating agents in the same way as the parental SSK cells. The radiation sensitivity is unchanged in all cell lines, even after activation of cAMP or cGMP. These results suggest that specific DNA repair pathways are altered by radiation but affected only in cisplatin damage repair, which is regulated by cGMP. Although there is ample cooperativity and interaction between the cAMP- and the cGMP-dependent transduction pathways, specific substrate binding by cGMP appears to play an important role in radiation-induced cisplatin resistance.


British Journal of Cancer (1995) 72, 287-292
© 1995 Stockton Press All rights reserved 0007-0920/95 $12.00

### Reversal of radiation-induced cisplatin resistance in murine fibrosarcoma cells by selective modulation of the cyclic GMP-dependent transduction pathway

H Eichholtz-Wirth

*GSF-Institut für Strahlenbiologie, D-85758 Neuherberg and Strahlenbiologisches Institut der Universität, Schillerstrasse 42, D-80336 Munich, Germany.*

**Summary** Cisplatin resistance, induced in murine fibrosarcoma cells (SSK) *in vitro* or *in vivo* by low-dose irradiation, can be overcome by activation of the cyclic GMP(cGMP)-dependent transduction pathway. This is mediated either by stimulating cGMP formation with sodium nitroprusside or by replacing cGMP with a selective activator of the cGMP-dependent protein kinase, 8-bromo-cGMP. The cyclic AMP-dependent transduction pathway is not involved in cisplatin resistance. Instead, activation of cAMP sensitises both parental and resistant SSK cells equally to the action of cisplatin. There is a 1.8 to 2.5-fold increase in drug toxicity, depending on the activating agent. Enhancement of cisplatin sensitivity is induced by specific inhibition of cAMP hydrolysis, increase in cAMP formation or by increasing the activation potential to cAMP-dependent protein kinase by specific cAMP analogues. Cells that have lost cisplatin resistance respond to cGMP- or cAMP-elevating agents in the same way as the parental SSK cells. The radiation sensitivity is unchanged in all cell lines, even after activation of cAMP or cGMP. These results suggest that specific DNA repair pathways are altered by radiation but affected only in cisplatin damage repair, which is regulated by cGMP. Although there is ample cooperativity and interaction between the cAMP- and the cGMP-dependent transduction pathways, specific substrate binding by cGMP appears to play an important role in radiation-induced cisplatin resistance.

**Keywords:** cisplatin resistance; GMP-dependent protein kinase; irradiation

Recently, we reported that cisplatin resistance after low-dose irradiation is associated with alterations in the cAMP-dependent signal transduction pathway in murine SSK fibrosarcoma cells (Eichholtz-Wirth and Hietel, 1994). In the presence of the non-selective phosphodiesterase inhibitor 3-isobutyl-1-methylxanthine (IBMX), cisplatin sensitivity could be restored in the resistant SSK cells, whereas only little effect was observed in the parental SSK cells. This implies that cAMP or cGMP is the target in the cascade of signal transduction.

The two second messengers cAMP and cGMP are key regulatory molecules with specific cellular functions. Cyclic AMP mediates its effects through binding to its main receptor protein, cAMP-dependent protein kinase (cAMP-PK) which exists in different subunits with various isoforms (Ogreid *et al.*, 1989; Cho-Chung, 1990). These isozymes differ in tissue distribution, cAMP dissociation rate and analogue specificity. They may be selectively modulated by autophosphorylation, by cross-activation with cGMP binding sites or by translocation (Jiang *et al.*, 1992). The cAMP-dependent protein kinase may activate transcription factors, which affect – by positive or negative regulation – DNA-binding activity, the transcriptional activity or the subcellular localisation of transcription factors. One of the best studied transcription factors is the cAMP response element-binding protein CREB (Karin, 1994), which has also been associated with drug-resistance (Rohlff *et al.*, 1993*a*).

The cGMP is less widely abundant and distributed than cAMP, but also has a diversity of cellular effects. cAMP-PK and cGMP-PK are evolutionarily related enzymes which are homologous in structure and function and similar in substrate specificity (Francis and Corbin, 1994). There are three major receptor proteins in mammalian cells: the cGMP-gated cation channel, cGMP-stimulated and inhibited phosphodiesterase (PDE) and cGMP-dependent protein kinase, cGMP-PK (Hofmann *et al.*, 1992). These receptor proteins differ widely in specific cells and they may be positively or negatively affected by cGMP (Lincoln and Cornwell, 1993). Autophosphorylation of type I cGMP-PK in the presence of cAMP increases basal activity and affinity for cAMP and decreases cooperativity between cGMP binding sites.

One of the major mechanisms of cGMP-PK-catalysed phosphorylation in most tissues is the regulation of intracellular $Ca^{2+}$ levels. The cGMP-dependent signal transduction cascade is not well understood and may involve phosphorylation of other cGMP receptors, such as the high mobility group protein (HMG), which has been associated with cisplatin resistance (Walton *et al.*, 1982; Chao *et al.*, 1991). IBMX inhibits the hydrolysis of both the short-lived second messengers cAMP and cGMP.

To analyse further the mechanism of radiation-induced cisplatin resistance in our SKK cells, it therefore has to be determined whether the cAMP-dependent or the cGMP-dependent signal transduction pathway is affected. Owing to ample cooperativity between these two pathways, it might be difficult to differentiate between them. Secondly, it would be interesting to know whether the observed changes in cisplatin sensitivity between parental and drug resistant cells can be attributed to any of the following three levels of signal transduction: (1) cyclic nucleotide hydrolysis, (2) cyclic nucleotide synthesis, and/or (3) cyclic nucleotide binding to the protein kinase.

(1) If cisplatin resistance results from altered *cyclic nucleotide hydrolysis*, inhibition would have to be demonstrated by selective isozyme inhibition against one of the five specific phosphodiesterase isozyme families. These are differently expressed and regulated in different cell types. The xanthine dervative IBMX is a non-specific PDE inhibitor which also acts as a potent antagonist for adenosine receptors (Beavo and Reifsnyder, 1990).
(2) Cyclic nucleotides may be affected at the level of *synthesis* through modification of type-specific adenyl cyclases (Souness *et al.*, 1990; Yoshimura and Cooper, 1993). These isozymes may have distinct cellular distribution and may be differentially stimulated by $Ca^{2+}$ and protein kinase C (PKC) (Jakobowitz *et al.*, 1993).
(3) Finally, the cyclic nucleotide *binding* to the binding domains of the protein kinases may be modulated.

Correspondence: H Eichholtz-Wirth, Strahlenbiologisches Institut der Universität, Schillerstrasse 42, D-80336, Munich, Germany
Received 17 January 1995; revised 13 March 1995; accepted 16 March 1995

Abnormal expression of type I and type II cAMP-PK has been described with changes in cAMP receptor isoforms (Ogreid *et al.*, 1989; Cho-Chung, 1990). This has been associated with malignant transformation, differentiation and cell growth (Ally *et al.*, 1988; Rohlff *et al.*, 1993*a*) as well as drug resistance (Nishio *et al.*, 1992; Rohlff *et al.*, 1993*b*). Studies on cyclic nucleotide substituents have revealed differing binding affinities and/or activation capacities for the regulatory domains of the various isozymes for the cAMP-dependent and the cGMP-dependent protein kinase pathway (Cho-Chung, 1990; Hofmann *et al.*, 1992).

The purpose of the present study was to obtain more information on the mechanisms involved in radiation-induced cisplatin resistance in SSK cells. In particular, which of the cyclic nucleotide-dependent signal transduction pathways is affected and at what level should be determined. This includes the question of how the alteration can be overcome by specific agonists or inhibitors.

### Materials and methods

### *Materials*

The following drugs and chemicals were used: cisplatin solution from Medac, Hamburg; Br-cGMP from Biolog, Bremen; Eagle's minimum essential medium (MEM) from Serva, Heidelberg; bycomycin from Byk Gulden, Konstanz; newborn calf serum from C.C.Pro, Karlsruhe; all other chemicals were purchased from Sigma Chemie, Deisenhofen. The substances were dissolved as follows: sodium nitroprusside (SNP) (10 mM), isoprenaline (50 mM), cAMP/cGMP analogues (1 mM) in water; 12-*O*-tetradecanoyl phorbol 13-acetate (TPA) (1 mM), staurosporine (0.2 mM) in dimethyl sulphoxide (DMSO); the PDE inhibitors (10 mM) in alcohol/water; stock solutions were stored at $-20°C$ for short periods of time, if necessary.

### *Cell lines*

Induction of cisplatin resistance after low-dose radiation and cisplatin conditioning was described in detail recently (Eichholtz-Wirth and Hietel, 1994). Briefly, SSK-R3 and SSK-R5 cells were derived from mouse fibrosarcoma cells (SSK) after low-dose irradiation *in vitro* ($5 \times 2$ Gy over 7 days). These cells exhibited a transient cisplatin resistance. After loss of drug resistance between passages 4 and 6 following irradiation, SSK-R3 but not SSK-R5 cells were submitted once to a conditioning cisplatin treatment ($0.5\ \mu g\ ml^{-1}$, 48 h). Cisplatin resistance was thus restored and maintained for at least 25 passages, equivalent to about 75 cell cycles. The same drug exposure alone without preirradiation did not generate cisplatin resistance.

The growth characteristics of the resistant SSK cells are similar to those of the parental SSK cells (for details see Eichholtz and Hietel, 1994), except for slightly longer doubling times (12–15 h as compared with 11–13 h for SSK cells). Glutathione (GSH) content is not significantly different, while cadmium chloride sensitivity, which is an indirect measure of metallothioneins (Eichholtz-Wirth *et al.*, 1993), is reduced in SSK-R3 cells by a factor of 1.6.

### *Cell culture*

All cell lines were grown as monolayer cultures in Eagle's MEM, supplemented with 10% newborn calf serum, 0.01% bycomycin and 0.035% sodium bicarbonate and maintained at 37°C at pH 7.4 in a controlled carbon dioxide atmosphere (3–3.5% carbon dioxide).

### *Determination of drug and radiation sensitivity*

To establish cisplatin survival curves, exponentially growing cells were appropriately diluted and allowed to attach to the glass surface overnight. Exposure to cisplatin in combination with or without other drugs was carried out in culture medium at varying cisplatin concentrations. The drugs were freshly diluted in Hanks' solution and added to the culture medium. After the allotted exposure time the medium was decanted, the cells rinsed with Hanks' solution and fresh culture medium was added.

For combined treatment experiments, using cisplatin (or radiation) and a second drug, non-toxic or low-toxic drug concentrations were used for the second drug, resulting in a cell survival of 0.8–1.0 for the second drug alone; in any case, the surviving fractions were corrected for the toxicity of this second drug alone.

To generate radiation survival curves, cells were exposed to graded single doses of γ-rays from a Gammacell 40 caesium-137 source at a dose rate of 1.2 Gy $min^{-1}$. For combined treatment, the cells were exposed to the drugs directly before irradiation. After 7–9 days' incubation, all flasks were scored for colonies of 50 or more cells. The surviving fraction (SF) was corrected for the plating efficiency of untreated cells.

### *Data and figures*

All figures shown represent the combined results of at least three independent experiments. Enhancement factors ($E_F$) and resistance factors ($R_F$) were calculated from $IC_{10}$ values of the survival curves. (The $IC_{10}$ is the drug concentration at a given exposure time necessary to reduce the cell survival to 10%.) Thus $E_F$ is defined as cisplatin exposure alone divided by cisplatin in the presence of a second drug. $R_F$ is defined as the $IC_{10}$ of resistant cells divided by the $IC_{10}$ of parental cells.

Standard deviation bars are shown except where the error is less or equal to the symbol size. *P*-values are given for statistical significance between various treatment procedures.

### Results

Survival curves of drug-resistant SSK-R3 cells and parental SSK cells as a function of increasing cisplatin concentrations for 1 h are shown in Figure 1. There is a 1.9-fold cisplatin resistance in SSK-R3 cells ($R_F = 1.9 \pm 0.15$) compared with the parental SSK cells.

If cisplatin treatment is carried out in the presence of isoprenaline, an enhancer of adenyl cyclase, cell survival is reduced. However, this sensitising effect is observed in the sensitive as well as in the resistant cells ($E_F = 2.1$ for SSK-R3 cells and 1.8 for SSK cells, Figure 1a). All cisplatin-sensitising agents that were used in the following experiments were tested to give the same modification of cisplatin cytotoxicity in parental and resistant SSK cells at various concentrations. The concentrations used were non-toxic or only slightly toxic when given alone ($SF > 0.8$). There is a similar increase in cell kill if cisplatin exposure is combined with 8-Br-cAMP (Figure 1b). This cAMP analogue is an activator of protein kinase A, with increased hydrolytical stability and membrane permeability as compared with cAMP. The enhancement factors of 2.2 and 2.3 for SSK-R3 and SSK cells indicate that the cAMP-dependent transduction pathway is involved in the response to the cisplatin damage. However, this does not explain the mechanism responsible for drug resistance as was anticipated from our previous results following IBMX treatment. These results demonstrated that exposure of the resistant SSK cells to cisplatin in the presence of IBMX restored drug sensitivity. Since this non-selective PDE inhibitor also affects the less common cGMP-dependent transduction pathway, the combined action of the guanylate cyclase activator SNP and cisplatin was studied. Figure 2a shows cell survival as a function of various cisplatin concentrations in the presence or absence of 10 μM SNP. This stimulator of cGMP formation increases cisplatin cytotoxicity in both cell lines and selectively restores cisplatin sensitivity in the resistant SSK-R3 cells ($E_F = 2.9$ and 1.6 for SSK-R3 and SSK cells respec-

**Figure 1** Surviving fraction of parental SSK (triangles) and cisplatin-resistant SSK-R3 cells (circles) as a function of various cisplatin concentrations for 1 h (**a**) Survival in the presence (closed symbols) or absence (open symbols) of the cAMP-modulating agent isoprenaline (5 μM). (**b**) Survival in the presence (closed symbols) or absence (open symbols) of the cAMP analogue 8-Br-cAMP (5 μM). Data points are means of at least three single experiments ± s.d.

**Figure 2** Surviving fraction of parental SSK (triangles) and cisplatin-resistant SSK-R3 cells (circles) as a function of various cisplatin concentrations for 1 h. (**a**) Survival in the presence (closed symbols) or absence (open symbols) of the cGMP-stimulating agent SNP (10 μM.) (**b**) Survival in the presence (closed symbols) or absence (open symbols) of the cGMP analogue 8-Br-cGMP (5 μM). Data points are means of at least three single experiments ± s.d.

**Table I** Effects of selective PDE inhibitors on cisplatin sensitivity in SSK and SSK-R3 cells

| *PDE inhibitor* | *SSK cells* $IC_{10}$[a] | $E_F$[b] | *SSK-R3 cells* $IC_{10}$[a] | $E_F$[b] |
|---|---|---|---|---|
| None (cisplatin alone) | 3.0 | 1.0 | 5.5 | 1.0 |
| Amrinone | 1.3 | 2.3* | 2.6 | 2.1* |
| Propentofylline | 1.6 | 1.9* | 2.9 | 1.9* |
| Dipyridamole | 1.8 | 1.7* | 2.9 | 1.8* |

[a]Data are derived from the survival curves after 1 h drug exposure (5 μM PDE inhibitor combined with varying cisplatin concentrations); mean of at least three experiments. [b]Enhancement factors ($E_F$) are determined from the $IC_{10}$ values (μM): cisplatin alone *vs* cisplatin combined with a PDE inhibitor. *Not significantly different between SSK and SSK-R3 cells (*P*-values > 0.05).

**Table II** Influence of various cAMP analogues on cisplatin sensitivity in SSK and SSK-R3 cells

| *cAMP analogue* | *SSK cells* $IC_{10}$[a] | $E_F$[b] | *SSK-R3 cells* $IC_{10}$[a] | $E_F$[b] |
|---|---|---|---|---|
| None (cisplatin alone) | 3.0 | 1.0 | 5.5 | 1.0 |
| 8-Br-cAMP (5 μM) | 1.8 | 1.7* | 3.0 | 1.8* |
| 8-cpt-cAMP (50 μM) | 1.6 | 1.9** | 2.5 | 2.2** |
| 8-Cl-cAMP (5 μM) | 1.4 | 2.1* | 2.4 | 2.3* |

[a]Data are derived from survival curves after 1 h drug exposure; mean of at least three separate experiments. [b]Enhancement factors ($E_F$) are determined from the $IC_{10}$ values (μM): cisplatin alone *vs* cisplatin combined with a cAMP analogue. *Not significantly different between SSK and SSK-R3 cells (*P*-values > 0.05). ***P* < 0.05.

tively). There is also differential enhancement in cisplatin toxicity when the cells are exposed in combination with the cGMP agonist 8-Br-cGMP (Figure 2b; $E_F = 2.5$ and 1.7 for SSK-R3 and SSK cells respectively).

Selective inhibition of the various PDE isoforms is presented in Table I. Amrinone is efficient against the cGMP-inhibited PDE III. Propentofylline inhibits cAMP-specific PDE IV and, to a lesser extent, the calcium–calmodulin-dependent isoform I. Dipyridamole, the cGMP-specific inhibitor of type V PDE, is also a potent inhibitor of adenosine transport. All these agents enhance cisplatin toxicity, but there is no significant difference between sensitive and resistant SSK cells.

More widely used cyclic nucleotide substituents, which have been associated with drug resistance and inhibition of proliferation, also increase cisplatin sensitivity in both cell lines, but do not play any specific role in radiation-induced cisplatin resistance in SSK cells (Table II). 8.Cl-cAMP enhances cisplatin sensitivity slightly more than 8-Br-cAMP, but there is no significant difference between the enhancement ratios in SSK cells compared with the SSK-R3 cells ($P$-values > 0.05). Only 8-$p$-chlorophenylthio)-cAMP (8-cpt-cAMP) is slightly more effective in the resistant cells at a concentration of 50 μM.

In contrast to the enhancement of cisplatin cytotoxicity by various agonists of the cAMP and cGMP-dependent pathways, cisplatin sensitivity is unchanged upon activation of the more prominent protein kinase C pathway. Neither the PKC activator TPA nor the PKC inhibitor staurosporine exerts any influence on SSK-R3 or SSK cell survival in combination with cisplatin (Figure 3).

As has already been seen in cisplatin-resistant SSK cells that were induced by high-dose irradiation (Eichholtz-Wirth *et al.*, 1993), the radiation sensitivity was again unchanged in SSK-R3 and SSK cells (Figure 4). When the cells were irradiated immediately after 1 h SNP treatment as described for the corresponding cisplatin experiments, there was no alteration in radiosensitivity.

SSK-R5 cells which had been irradiated but not cisplatin conditioned as the SSK-R3 cells exhibited a short, transient cisplatin resistance only during 20–30 cell cycles. When they have lost their cisplatin resistance, these cells respond to cGMP agonists in a similar way as do the parental SSK cells (Figure 5). There is no significant difference in the enhancement factors.

**Figure 4** Radiation sensitivity of SSK (triangles) and SSK-R3 (circles) cells in the presence (closed symbols) or absence (open symbols) of 10 μM SNP. One typical experiment is shown.

### Discussion

The present data demonstrate that radiation-induced cisplatin resistance in murine fibrosarcoma SSK-R3 cells can be overcome by stimulation of the cGMP-dependent transduction pathway. This activation may be achieved with similar effectiveness by enhancing cGMP formation with SNP or by increasing cGMP binding by replacing cGMP with the cGMP analogue 8-Br-cGMP.

This analogue was selected because of its higher specificity and lipophilicity as compared with cGMP; it is hydrolysed only to a small extent by cyclic nucleotide phosphodiesterases and it is a poor activator of the cAMP-dependent protein kinase (Butt *et al.*, 1992). This cGMP agonist has a higher potency to activate cGMP-PK than cGMP ($K'_a$ for the regulatory subunit type Iα = 4.3) with preferential binding to the slow exchanging site (Sekhar *et al.*, 1992).

SNP enhances cGMP by stimulation of guanylate cyclase; there are conflicting findings as to the activation of the cGMP-dependent kinase by SNP (Lincoln and Keely, 1981; Geiger *et al.*, 1992), which might be affected differently in various cell types.

Using these two cGMP-selective agents, it is possible to distinguish between the cAMP-dependent and the cGMP-

**Figure 5** Cell survival as a function of cisplatin concentration in the presence (closed symbols) or absence (open symbols) of 10 μM SNP. Triangles, parental SSK cells; circles, cisplatin-resistant SSK-R3 cells; squares, SSK-R5 cells, which had lost cisplatin resistance. Data points are means of at least three single experiments ± s.d.

**Figure 3** Cell survival of SSK cells (triangles) and SSK-R3 cells (circles) as a function of a 1 h cisplatin exposure alone (open symbols) or in combination with either (**a**) the PKC inhibitor staurosporine (10 nM, closed symbols) or (**b**) the PKC activator TPA (100 nM, closed symbols). Data points are means of at least three single experiments ± s.d.

dependent transduction pathways. Selective restoration of cisplatin sensitivity in the resistant SSK-R3 cells is observed only after stimulation of the cGMP-dependent pathway, and not after activation of the cAMP-dependent transduction pathway. This differential effect of cGMP-enhancing agents on sensitive and resistant SSK cells correlates with loss of drug resistance. In addition, the enhancement of cisplatin cytotoxicity is unlikely to be due to the expression of cell damage by free radicals, since the cGMP analogue 8-Br-cGMP is structurally different from the nitric oxide-generating drug SNP. The alteration in the cGMP pathway in SSK-R3 cells does not seem to be associated with enhanced hydrolysis of one of the phosphodiesterase isozymes, although the diversity of isozymes may reveal high variation in regulatory interactions. Moreover, cGMP exerts one of its main regulatory functions on the receptor protein phosphodiesterase (Lincoln and Cornwell, 1993). In SSK and SSK-R3 cells, specific inhibition of the PDE isozymes increased cisplatin sensitivity in both cell lines, without differential effects on SSK-R3 cells.

Mechanisms other than recognition of specific motifs may contribute to selective cGMP-mediated protein phosphorylation. Little is known about protein substrates for cGMP-PK and their localisation. One of the possible substrates may include the HMG proteins, which are specifically phosphorylated by cyclic nucleotide-dependent protein kinases (Walton *et al.*, 1982). These transcription factors regulate, at least in part, the relative sensitivity of cells to cisplatin. They recognise the distortions in DNA structure resulting from cisplatin–adduct formation, preferentially bind to cisplatin-damaged DNA and allow increased accessibility of DNA repair enzymes (Chao *et al.*, 1991; Hughes *et al.*, 1992). In cisplatin-resistant HeLa cells overexpression of damage recognition proteins has been suggested to be responsible for altered DNA repair and emergence of drug resistance (Chao *et al.*, 1991).

The recently described absence of cross-resistance towards other cytostatic drugs in resistant SSK cells (Eichholtz-Wirth and Hietel, 1994) would also argue for cisplatin-specific transcription factors to be involved in altered damage recognition and repair in SSK-R3 cells. This raises the possibility that these widely distributed chromatin proteins are substrates for cGMP or cGMP-PK in SSK-R3 cells.

In most reports on the involvement of signal transduction pathways in drug resistance, alterations of the more prominent protein kinase C pathway have been observed (Isonishi *et al.*, 1990; Basu and Lazoa, 1992; Rubin *et al.*, 1992). In SSK and SSK-R3 cells, neither PKC activation by TPA nor PKC inhibition by staurosporine had any effect on cisplatin-dependent survival curves. Only few studies have reported drug resistance to be associated with changes in the cyclic nucleotide-dependent signal transduction. Abraham *et al.* (1987) reported cAMP-dependent protein kinase to be involved in regulating resistance in CHO cells to a variety of drugs. Mutant cells with defective regulatory subunit RI for cAMP-PK were drug sensitive, whereas revertants simultaneously regained normal drug resistance and cAMP sensitivity. Doxorubicin resistant HL-60 cells could be selectively sensitised by 8-Cl-cAMP with complete down-regulation of nuclear type I PKA (Rohlff *et al.*, 1993*a*). Concurrently, there was a reduction in the DNA-binding activity of the transcription factors CREB, AP-1 and AP-2, whose activities were markedly enhanced in the resistant cells. In contrast to our results, drug cytotoxicity could be enhanced in cisplatin-resistant human ovarian carcinoma cells 2008 by treatment with IBMX, but not with the adenyl cyclase agonist forskolin (Mann *et al.*, 1991). This cyclic nucleotide-dependent cisplatin response was later shown to be associated with alterations of microtubules (Christen *et al.*, 1993). In SSK cells this effect is unlikely to mediate cisplatin resistance, since membrane-sensitising agents such as vincristine or the calcium antagonist nifedipine did not exhibit a differential response in sensitive and resistant SSK cells (Eichholtz-Wirth and Hietel, 1994).

The data suggest that, in SSK cells, the cAMP-dependent transduction pathway is not associated with cisplatin resistance, but rather confers increased drug toxicity in both cell lines. This is demonstrated by combined treatment of cisplatin with cAMP analogues or with isoprenaline, which stimulates cAMP formation by activation of adenylate cyclase (Hall *et al.*, 1992). Of the various cAMP analogues described in the literature, we used 8-Br-cAMP, which has a lower activation constant for PKA and slightly greater lipophilicity than cAMP (Sandberg *et al.*, 1991) and, because of its specificity, allows a distinction between the cAMP- and the cGMP-dependent transduction pathway. 8-cpt-cAMP, which has been extensively studied as a selective activator of cAMP-dependent protein kinase, is also a potent inhibitor of the cyclic GMP-specific phosphodiesterase type V (Connolly *et al.*, 1992) and stimulates both cGMP-PK- and cAMP-PK-mediated protein phosphorylation (Sandberg *et al.*, 1991). 8-Cl-cAMP was reported by Cho-Chung (1990) to be an excellent site-selective cAMP analogue owing to high-affinity binding to RII but not RI and low activation constant for RII. This analogue, however, might act through its adenosine metabolite (van Lookeren Campagne *et al.*, 1991; Lange-Carter *et al.*, 1993). 8-Cl-cAMP and 8-cpt-cAMP are therefore not good choices as site-selective cAMP analogues when the effects of cGMP and cAMP are to be distinguished. The changes that account for the observed cisplatin resistance involve steps in the signal transduction pathway distinct from those that participate in the cAMP-dependent increase in cisplatin sensitivity.

Although there is a close relationship between the cGMP-dependent and the cAMP-dependent pathway, with ample cooperativity and interactions between these two pathways, our results suggest that a specific substrate is phosphorylated only by the cGMP-dependent pathway, which is a mediator of cisplatin resistance. The increase in cisplatin sensitivity after stimulation of the cAMP-dependent pathway which is seen in resistant and sensitive SSK cells may be related to activation of transcription factors, such as CREB or AP-1 or with altered expression of type I and type II cAMP-dependent protein kinase, as described above.

Radiation rapidly activates early response gene signalling cascades (Wilson *et al.*, 1993), however altered response to ionising radiation has been reported only after inhibition of protein kinase C (Hallahan *et al.*, 1992), but not in relation to cyclic nucleotides. In SSK-R3 cells, the radiosensitivity is not significantly changed compared with the parental cells, with or without additional SNP treatment. A possible explanation is that cisplatin-specific DNA repair pathways are altered in the resistant cells. These changes are probably not involved in the damage repair after ionising radiation and are regulated by a cGMP-dependent pathway.

The present results demonstrate that radiation-induced cisplatin resistance in SSK-R3 cells can be overcome by stimulation of the cGMP-dependent transduction pathway. Enhancement of the closely related cAMP-dependent pathway does not correlate with cisplatin resistance; however, it markedly increases cisplatin cytotoxicity in sensitive and resistant SSK cells. It will be of interest now to determine the cGMP receptor in SSK-R3 cells and to see whether radiation-induced alterations of the cGMP-dependent transduction pathway are also observed in different cell systems.

**Abbreviations**

Cisplatin, cis-diamminedichloroplatinum (II); cAMP, cyclic AMP; cGMP, cyclic GMP; cAMP-PK, cAMP-dependent protein kinase; cGMP-PK, cGMP-dependent PK; 8-Br-cGMP, 8-bromo-cGMP; 8-Br-cAMP, 8-bromo-cAMP; 8-Cl-cAMP, 8-chloro-cAMP; 8-cpt-cAMP, 8-(*p*-chlorophenylthio)-cAMP; RI, RII, regulatory subunits I and II of cAMP-PK; PDE, cyclic nucleotide phosphodiesterase; PDE I, $Ca^{2+}$/calmodulin-stimulated PDE; PDE II, cGMP-stimulated PDE; PDE III, cGMP-inhibited PDE; PDE IV, cAMP-specific PDE;

PDE V, cGMP-specific PDE; IBMX, 3-isobutyl-1-methylxanthine; PKC, protein kinase C; GSH, glutathione; SNP, sodium nitroprusside; TPA, 12-*O*-tetradecanoyl phorbol 13-acetate; SF, surviving fraction; $E_F$, enhancement factor; $R_F$, resistance factor.

**Acknowledgement**
I would like to thank Dr Wolfgang Dostmann, Pharmakologisches Institut, TU Munich, for valuable discussion and Miss Renate Hintermaier for skilful technical assistance.

**References**

ABRAHAM I, HUNTER RJ, SAMPSON KE, SMITH S, GOTTESMAN MM AND MAYO JK. (1987). Cyclic AMP-dependent protein kinase regulates sensitivity of cells to multiple drugs. *Mol. Cell. Biol.*, **7**, 3098–3106.

ALLY S, TORTORA G, CLAIR T, GRIECO D, MERLO G, KATSAROS D, OGREID D, DOSKELAND SO, JAHNSEN T AND CHO-CHUNG YS. (1988). Selective modulation of protein kinase isozymes by the site-selective analog 8-chloroadenosine 3′,5′-cyclic monophosphate provides a biological means for control of human colon cancer cell growth. *Proc. Natl Acad. Sci. USA*, **85**, 6319–6322.

BASU A AND LAZO JS. (1992). Sensitization of human cervical carinoma cells to cis-diamminedichlorplatinum(II) by Bryostatin. *Cancer Res.*, **52**, 3119–3124.

BEAVO JA AND REIFSNYDER DH. (1990). Primary sequence of cyclic nucleotide phosphodiesterase isozymes and the design of selective inhibitors. *Trends Pharmacol. Sci.*, **11**, 150–155.

BUTT E, NOLTE C, SCHULZ S, BELTMAN J, BEAVO JA, JASTORFF B AND WALTER U. (1992). Analysis of the functional role of cGMP-dependent protein kinase in intact human platelets using the specific activator 8-para-chlorophenylthio-cGMP. *Biochem. Pharmacol.*, **43**, 2591–2600.

CHAO CCK, HUANG SL, LEE LY AND CHAO SL. (1991). Identification of inducible damage recognition proteins that are overexpressed in HeLa cells resistant to cis-diamminedichlorplatinum(II). *Biochem. J.*, **277**, 875–878.

CHO-CHUNG YS. (1990). Role of cyclic AMP receptor proteins in growth, differentiation, and suppression of malignancy: new approaches to therapy. *Cancer Res.*, **50**, 7093–7100.

CHRISTEN RD, JEKUNEN AP, JONES JA, THIEBAU F, SHALINSKY DR AND HOWELL SB. (1993). In vitro modulation of cisplatin accumulation in human ovarian carcinoma cells by pharmacologic alteration of microtubules. *J. Clin. Invest.*, **92**, 431–440.

CONOLLY BJ, WILITS PB, WARRINGTON BH AND MURRAY KJ. (1992). 8-(chlorophenyl)thio-cyclic AMP is a potent inhibitor of the cyclic GMP-specific phosphodiesterase (PDE $V_A$). *Biochem Pharmacol.*, **44**, 2303–2306.

EICHHOLTZ-WIRTH H AND HIETEL B. (1994). Cisplatin resistance in mouse fibrosarcoma cells after low-dose irradiation *in vitro* and *in vivo*. *Br. J. Cancer*, **70**, 579–584.

EICHHOLTZ-WIRTH H, REIDEL G AND HIETEL B. (1993). Radiation-induced transient cisplatin resistance in murine fibrosarcoma cells associated with elevated metallothionein content. *Br. J. Cancer*, **67**, 1001–1006.

FRANCIS SH AND CORBIN JD. (1994). Structure and function of cyclic nucleotide-dependent protein kinase. *Annu. Rev. Physiol.*, **56**, 237–272.

GEIGER J, NOLTE C, BUTT E, SAGE SO AND WALTER U. (1992). Role of cMP and cGMP-dependent protein-kinase in nitrovasodilator inhibition of agonist-evoked calcium elevation in human platelets. *Proc. Natl Acad. Sci. USA*, **89**, 1031–1035.

HALL IP, WIDDOP S, TOWNSEND P AND DAYKIN K. (1992). Control of cyclic AMP levels in primary cultures of human tracheal smooth muscle cells. *Br. J. Pharmacol.*, **107**, 422–428.

HALLAHAN DE, VIRUDACHALAM S, SCHWARTZ JL, PANJA N, MUSTAFI R AND WEICHSELBAUM RR. (1992). Inhibition of protein kinases sensitizes human tumor cells to ionizing radiation. *Radiat. Res.*, **129**, 345–350.

HOFMANN F, DOSTMANN W, KEILBACH A, LANDGRAF W AND RUTH P. (1992). Structure and physiological role of cGMP-dependent protein kinase. *Biochim. Biophys. Acta*, **1135**, 51–60.

HUGHES EN, ENGELSBERG BN AND BILLINGS PC. (1992). Purification of nuclear proteins that bind to cisplatin-damaged DNA. *J. Biol. Chem.*, **267**, 13520–13527.

ISONISHI S, ANDREWS PA AND HOWELL SB. (1990). Increased sensitivity to cis-diamminedichlorplatinum(II) in human ovarian carcinoma cells in response to treatment with 12-O-Tetradecanoylphorbol 13-acetate. *J. Biol. Chem.*, **265**, 3623–3627.

JACOBOWITZ O, CHEN J, PREMONT RT AND IYENGAR R. (1993). Stimulation of specific types of $G_S$-stimulated adenyl cyclases by phorbol ester treatment. *J. Biol. Chem.*, **268**, 3829–3832.

JIANG H, COLBRAN JI, FRANCIS SH AND CORBIN JD. (1992). Direct evidence for cross-activation of cGMP-dependent protein kinase by cAMP in pig coronary arteries. *J. Biol. Chem.*, **267**, 1015–1019.

KARIN M. (1994). Signal transduction from the cell surface to the nucleus through the phosphorylation of transcription factors. *Curr. Opin. Cell Biol.*, **6**, 415–426.

LANGE-CARTER CA, VUILLEQUEZ JJ AND MALKINSON AM. (1993). 8-Chloroadenosine mediates 8-chloro-cyclic AMP-induced down-regulation of cyclic AMP-dependent protein kinase in normal and neoplastic mouse lung epithelial cells by a cyclic AMP-independent mechanism. *Cancer Res.*, **53**, 393–400.

LINCOLN TM AND CORNWELL TL. (1993). Intracellular cyclic GMP receptor protein. *FASEB J.*, **7**, 328–338.

LINCOLN TM AND KEELY SL. (1981). Regulation of cardiac cyclic GMP-dependent protein kinase. *Biochim. Biophys. Acta*, **676**, 230–244.

MANN St. C, ANDREWS PA AND HOWELL St.B, (1991). Modulation of *cis*-diamminedichloroplatinum(II) accumulation and sensitivity by forskolin and 3-isobutyl-1-methylxanthine in sensitive and resistant human ovarian carcinoma cells. *Int. J. Cancer*, **48**, 866–872.

NISHIO K, MORIKAGE T, KUBOTA N, OHMORI T, TAKEDA Y, FUJIWARA Y, MIKI K, ABE K AND SAIJO N. (1992). Alteration of type II regulatory subunit of cAMP-dependent protein kinase in human cisplatin-resistant cells as a basis of collateral sensitivity to 8-chloro-cAMP. *Jpn J. Cancer Res.*, **83**, 754–760.

OGREID D, EKANGER R, SIVA RH, MILLER JP AND DOSKELAND SO. (1989). Comparison of the two classes of binding sites (A and B) of type I and type II cyclic-AMP-dependent protein kinases by using cyclic nucleotide analogs. *Eur. J. Biochem.*, **181**, 19–31.

ROHLFF C, CLAIR T AND CHO-CHUNG YS. (1993*a*). 8-Cl-cAMP induces truncation and down-regulation of the RIa subunit and up-regulation of the RIIb subunit of cAMP-dependent protein kinase leading to type II holoenzyme-dependent growth inhibition and differentiation of HL-60 leukemia cells. *J. Biol. Chem.*, **268**, 5774–5782.

ROHLFF C, SAFA B, RAHMAN A, CHO-CHUNG YS, KLECKER R AND GLAZER RI. (1993*b*). Reversal of resistance to adriamycin by 8-chloro-cyclic AMP in adriamycin-resistant HL-60 leukemia cells is associated with reduction of type I cyclic AMP-dependent protein kinase and cyclic AMP response element-binding protein DNA-binding activities. *Mol. Pharmacol.*, **43**, 372–379.

RUBIN E, KHARBANDER S, GUNJI H, WEICHSELBAUM R AND KUFE D. (1992). Cis-diamminedichlorplatinum(II) induces c-jun expression in human myeloid leukemia cells: potential involvement of protein kinase C-dependent signalling pathway. *Cancer Res.*, **52**, 878–882.

SANDBERG M, BUTT E, NOLTE C, FISCHER L, HALBRÜGGE M, BELTMAN J, JAHNSEN TJ, GENIESSER HG, JASTORFF B AND WALTER U. (1991). Characterization of Sp-5,6-dichloro-1-b-D-ribofuranosyl-benzimidazole-3′,5′-monophosphorothiate (Sp-5,6-dCl-cBiMPS) as a potent and specific activator of cyclic-AMP-dependent protein kinase in cell extracts and in intact cells. *Biochem. J.*, **279**, 521–527.

SEKHAR KR, HATCHETT RJ, WOLFE L, FRANCIS SH, WELLS JN, JASTORFF B, BUTT E, CHAKINALA MM AND CORBIN JD. (1992). Relaxation of pig coronory arteries by new and potent cGMP analogs that selectively activate type Ia, compared with type Ib, cGMP-dependent protein kinase. *Mol. Pharmacol.*, **42**, 103–108.

SOUNESS JE, DIOCEE BK, MARTIN W AND MOODIE SA. (1990). Pig aortic endothelial-cell cyclic nucleotide phosphodiesterases. *Biochem. J.*, **266**, 127–132.

VAN LOOKEREN CAMPAGNE MM, DIAZ FV, JASTORFF B AND KESSIN RH. (1991). 8-Chloroadenosine 3′,5′-monophosphate inhibits the growth of Chinese hamster ovary and Molt-4 cells through its adenosine metabolite. *Cancer Res.*, **51**, 1600–1605.

WALTON GM, SPIESS J AND GILL GN. (1982). Phosphorylation of high mobility group 14 protein by cyclic nucleotide-dependent protein kinases. *J. Biol. Chem.*, **257**, 4661–4668.

WILSON RE, TAYLOR SL, ATHERTON GT, JOHNSTON D, WATRES CM AND NORTON JD. (1993). Early response gene signalling cascades activated by ionising radiation in primary human B cells. *Oncogene*, **8**, 3229–3237.

YOSHIMURA M AND COOPER DMF. (1993). Type-specific stimulation of adenylcyclase by protein kinase C. *J. Biol. Chem.*, **268**, 4604–4607.